# Artificial Intelligence in Medical Imaging of the Breast

**DOI:** 10.3389/fonc.2021.600557

**Published:** 2021-07-22

**Authors:** Yu-Meng Lei, Miao Yin, Mei-Hui Yu, Jing Yu, Shu-E Zeng, Wen-Zhi Lv, Jun Li, Hua-Rong Ye, Xin-Wu Cui, Christoph F. Dietrich

**Affiliations:** ^1^ Department of Medical Ultrasound, China Resources & Wisco General Hospital, Academic Teaching Hospital of Wuhan University of Science and Technology, Wuhan, China; ^2^ Department of Medical Ultrasound, Hubei Cancer Hospital, Tongji Medical College, Huazhong University of Science and Technology, Wuhan, China; ^3^ Department of Artificial Intelligence, Julei Technology, Wuhan, China; ^4^ Department of Medical Ultrasound, The First Affiliated Hospital of Medical College, Shihezi University, Xinjiang, China; ^5^ Department of Medical Ultrasound, Tongji Hospital, Tongji Medical College, Huazhong University of Science and Technology, Wuhan, China; ^6^ Department Allgemeine Innere Medizin (DAIM), Kliniken Beau Site, Salem und Permanence, Bern, Switzerland

**Keywords:** artificial intelligence, machine learning, deep learning, breast, imaging

## Abstract

Artificial intelligence (AI) has invaded our daily lives, and in the last decade, there have been very promising applications of AI in the field of medicine, including medical imaging, *in vitro* diagnosis, intelligent rehabilitation, and prognosis. Breast cancer is one of the common malignant tumors in women and seriously threatens women’s physical and mental health. Early screening for breast cancer *via* mammography, ultrasound and magnetic resonance imaging (MRI) can significantly improve the prognosis of patients. AI has shown excellent performance in image recognition tasks and has been widely studied in breast cancer screening. This paper introduces the background of AI and its application in breast medical imaging (mammography, ultrasound and MRI), such as in the identification, segmentation and classification of lesions; breast density assessment; and breast cancer risk assessment. In addition, we also discuss the challenges and future perspectives of the application of AI in medical imaging of the breast.

## Introduction

Artificial intelligence (AI) is commonly defined as “a system’s ability to correctly interpret external data, to learn from such data, and to use those learnings to achieve specific goals and tasks through flexible adaptation”. Over the past 50 years, the dramatic growth of computer functions related to big data intrusion has pushed AI applications into new areas (1). Currently, AI can be found in voice recognition, face recognition, driverless cars and other new technologies, and the application of AI in medical imaging has gradually become an important topic of research. AI algorithms, particularly deep learning (DL) algorithms, have demonstrated remarkable progress in image recognition tasks. Methods ranging from convolutional neural networks to variational autoencoders have been found in a myriad applications in the medical image analysis field and have promoted the rapid development of medical imaging (2). AI has made great contributions to early detection, disease evaluation and treatment response assessments in the field of medical image analysis for diseases such as pancreatic cancer (3), liver disease (4), breast cancer (5), chest disease (6), and neurological tumors (7).

Approximately 2.1 million newly diagnosed cases of breast cancer occurred in 2018 worldwide, accounting for almost 1 in 4 of all cases of cancer among women (8). Breast cancer is the most frequently diagnosed cancer in most countries (154 of 185) and is the leading cause of death due to cancer in over 100 countries (9). Breast cancer has a marked impact on women’s physical and mental health, which seriously threatens women’s lives and health. The early screening and treatment of breast diseases have become major health problems in the world. The correct diagnosis, especially the early detection and treatment of breast cancer, has a decisive impact on the prognosis. The clinical cure rate of early breast cancer can reach more than 90%; in the middle stage, it is 50 - 70%, and in the late stage, the treatment effect is very poor. Currently, Mammography, ultrasound and MRI are invaluable screening and supplemental diagnostic tool for breast cancer, they also have become important means of detection, staging and efficacy evaluations and follow-up examinations of breast cancer (10).

At present, breast images are mainly read, analyzed and diagnosed by radiologists. Under a large and long-term workload, radiologists are more likely to misjudge images due to fatigue, resulting in a misdiagnosis or missed diagnosis, which can be avoided with AI. To avoid human errors, computer-aided diagnosis (CAD) has been implemented. In CAD systems, a suitable algorithm completes the processing and analysis of an image (11). The latest breakthrough is DL, especially convolutional neural networks (CNNs), which has made significant progress in the field of medical imaging (12). This article briefly introduces the background of AI and mainly reviews its application in breast mammography, ultrasound and MRI image analysis. This paper also discusses the prospects for the application of AI in medical imaging.

## Brief Overview of AI

AI refers to the ability of application machines to imitate humans or human brain functions to learn and solve problems (13). It has been more than 60 years since John McCarthy put forward the concept of AI in 1956. Over the past ten years, AI technology has made explosive progress. As a branch of computer science, it attempts to produce a new kind of intelligent machine that responds like a human brain; its application field is very wide and includes robots, image recognition, language recognition, natural language processing, data mining, pattern recognition and expert system, etc. (14, 15). In the medical field, AI can be applied to health management, clinical decision support, medical imaging, disease screening and early disease prediction, medical records/literature analysis, and hospital management, etc. AI can analyze medical images and information for disease screening and prediction and assist doctors in making diagnosis. In breast imaging, Al-antari MA et al. studied a complete integrated CAD system that can be used for detection, segmentation, and classification of masses in mammography in 2018, and its accuracy was more than 92% in all aspects (16). Alejandro Rodriguez-Ruiz et al. gathered 2654 exams and readings by 101 radiologists, using a trained AI system to score the possibility of cancer between 1 and 10, they found that using an AI score of 2 as the threshold could reduce the workload by 17%, which proved that the AI automatic preselection can significantly reduce the workload of radiologists (17).

Machine learning (ML) is one of the most important ways to realize AI. ML is divided into unsupervised and supervised. Unsupervised ML classifies the radiomics features without using any information provided by or determined by an available historical set of imaging data of the same kind of the one under investigation. Supervised ML methods are first trained by means of an available data archive, i.e. all parameters in the algorithm are tuned until the method provides an optimal trade-off between its ability to fit the training set and its generalization power when a new data example arrives. In the world of supervised ML, sparsity-enhancing regularization networks are able to make the prediction while, at the same time, identifying the extracted features that mostly impact such prediction (18). ML indicates those computational algorithms that utilize as input the image features extracted by radiomics in order to provide as output predictions concerning disease outcomes on follow-up, such as linear regression, K-means, decision trees, random forest, PCA (principal component analysis), SVM (support vector machine), and ANNs (artificial neural networks).

DL, one of the AI systems based on neural networks, is structured by building models that imitate the human brain and is currently considered to be the latest technology for image classification. Neural networks first simulate nerve cells and then try to simulate the human brain using a simulation model called a perceptron. A neural network consists of continuous layers, including the input layer, the hidden layer, and the output layer. The input layer can process multi-dimensional data, and the hidden layer includes a convolutional layer, pooling layer and fully connected layer. The feature map created in the convolutional layer is initially passed through a non-linear activation function, and this is then transferred to the pooling layer to enable down-sampling of the feature map. The output is then passed into the fully connected layer to classify the overall outcome, and the output layer directly outputs data analysis results. A multilayer perceptron is constructed by making and arranging layers with perceptrons in which all nodes in the model are fully connected together, thus solving more complex problems (19). The learning paradigm of CNNs also involves supervised learning and unsupervised learning; supervised learning refers to the training procedure in which the observed training data and the associated ground truth labels for that data (or sometimes referred to as “targets”) are both required for training the model. In contrast, unsupervised learning involves training data that has no diagnosis or normal/abnormal labels. Currently, supervised learning seems to be the most popular approach in image classification tasks (20).

## Applications of AI in Mammography

Mammography is one of the most widely used methods for breast cancer screening (21, 22). Mammography is a non-invasive detection method associated with relatively decreased pain, easy operation, high resolution, and good repeatability. The retained image can be compared before and after and is not limited by age or body shape. Mammography can detect breast masses that cannot be palpated by doctors and can reliably identify benign lesions and malignant tumors of the breast. Mammograms are currently acquired with full-field digital mammography (DM) systems and are provided in both for-processing (the raw imaging data) and for-presentation (a postprocessed version of the raw data) image formats (23, 24). To date, AI has been used to analyze mammography images in most studies mainly for the detection and classification of breast mass and microcalcifications, breast mass segmentation, breast density assessment, breast cancer risk assessment and image quality improvement.

### Detection and Classification of Breast Masses

Among the different abnormalities seen on mammograms, masses are one of the most common symptoms of breast cancer. It is difficult to detect and diagnose masses because of variation in the shape, size, and margins, especially in the presence of dense breasts. Therefore, mass detection is an essential step in CAD. Some studies proposed a Crow search optimization based intuitionistic fuzzy clustering approach with neighborhood attraction (CrSA-IFCM-NA), and it has been proven that CrSA-IFCM-NA effectively separated the masses from mammogram images and had good results in terms of cluster validity indices, indicating the clear segmentation of the regions (24). Others developed a complete integrated CAD system, which included a regional DL approach You-Only-Look-Once (YOLO) and a new deep network model full resolution convolutional network (FrCN) and a deep CNN, to detect, segment, and classify masses in mammograms and used the INbreast dataset to verify that quality detection accuracy reached 98.96%, effectively assisting radiologists make an accurate diagnosis (16, 25, 26).

### Detection and Classification of Microcalcifications

Breast calcifications are small spots of calcium salts in the breast tissue, and they appear as small white spots on mammography. There are two different types of calcifications: microcalcifications and macrocalcifications. Macrocalcifications are large and coarse and are mostly benign and age-related. Microcalcifications may be early signs of breast cancer, with sizes ranging from 0.1 mm to 1 mm, with or without visible masses (27). At present, several CAD systems have been developed to detect calcifications in mammography images. Cai H et al. developed a CNN model for the detection, analysis and classification of microcalcifications in mammography images and confirmed that the function of CNN model to extract images outperformed handcrafted features; they achieved a classification precision of 89.32% and a sensitivity of 86.89% by using filtered deep features that are fully utilized by the proposed CNN structure for traditional descriptors (28). Zobia Suhail et al. developed a novel method for the classification of benign and malignant microcalcifications using an improved Fisher linear discriminant analysis approach for the linear transformation of segmented microcalcification data in combination with a SVM variant to distinguish between the two classes; 288 region of interests (ROIs) (139 malignant and 149 benign) in the Digital Database for Screening Mammography (DDSM) were classified with an average accuracy of 96% (29). Jian W et al. developed a CAD system to detect breast microcalcifications based on dual-tree complex wavelet transform (DT-CWT) (30). To detect microcalcification in mammograms, Guo Y et al. proposed a new hybrid method *via* combining contourlet transform and non-linking simplified pulse-coupled neural network (31). An automatic neural network can automatically detect, segment and classify masses and microcalcifications in mammography, providing a reference for radiologists and significantly improving the work efficiency and accuracy of radiologists.

### Breast Mass Segmentation

The true segmentation of masses is directly related to the effective treatment of the patient. Some researchers used the method of fuzzy contours to automatically segment breast masses from mammograms and evaluated the ROIs extracted from the mini-MIAS database. The results showed that the average true positive rate was 91.12%, and the precision was 88.08% (32). Global segmentation of masses on mammograms is a complex process due to low-contrast mammogram images, irregular shapes of masses, spiculated margins, and the presence of intensity variations in pixels. Some used the mesh-free based radial basis function collocation approach for the evolution of a level set function for segmentation of the breast as well as suspicious mass regions. Then, an SVM classifier was used to classify the suspicious areas into abnormal and normal areas. The results showed that the sensitivity and specificity for the DDSM dataset were 97.12% and 92.43% respectively (33). Plane fitting and dynamic programming were applied to detect and classify breast mass in mammography, the accuracy of segmentation of breast lesions got improved greatly (34). The correct segmentation of breast lesions provides a guarantee for accurate disease classification and diagnosis (35). The use of an automatic image segmentation algorithm shows the application and potential of DL in precision medical systems.

### Breast Density Assessment

Breast density is a strong risk factor for breast cancer and is usually evaluated by two-dimensional (2D) mammograms. Women with higher breast density have a two- to six-fold higher risk of developing breast cancer than women with low breast density (36). Mammographic density has traditionally been assessed as the absolute or relative amount (as percentage of the total breast size) occupied by dense tissue, which appears on a mammographic images as white “cotton-like” patches (37). In the current context of breast density identification, accurate and consistent breast density assessment is highly desirable to provide clinicians and patients with more informed clinical decision-making support. Many studies have shown that AI technology can assist in the evaluation of mammographic breast density (BD). Mohamed AA et al. studied a CNN model based on the Breast Imaging Reporting and Data System (BI-RADS) for BD categorization and classified the density of large (i.e., 22000 images) DM datasets (i.e., “scattered density” and “heterogeneous density”); they showed that with an increase in training samples could achieve the highest the area under the receiver operating characteristic curve (AUC) of 0.94-0.98 (38). They also used a CNN model to show that radiologists mostly used a medial oblique (MLO) view rather than a head-to-tail (CC) view to determine the category of BD (39). Le Boulc’h M and others evaluated the agreement between DenSeeMammo (an AI-based automatic BD assessment software approved by the Food and Drugs Administration) and visual assessment by a senior and a junior radiologist, and found that the BD assessment between the senior radiologist and the AI model was basically the same on DM (weighted=0.79; 95%CI:0.73-0.84) (40). Lehman CD et al. developed and tested a DL model to assess BD by using 58 894 randomly selected digital mammograms, and implemented the model by using a deep CNN, ResNet-18, with PyTorch. And it is concluded that the agreement between density assessments with the DL model and those of the original interpreting radiologist was good (k = 0.67; 95% CI: 0.66, 0.68), and in the four-way BI-RADS categorization, 9729 of 10763 (90%; 95% CI: 90%, 91%) DL assessments were accepted by the interpreting radiologist (41). The assessment of MBD by AI can reduce the variation between radiologists, better predict the risk of breast cancer and provide a basis for further detection and treatment.

### Breast Cancer Risk Assessment

The high incidence and mortality of breast cancer are seriously threatening women’s physical and mental health. At present, there are many known risk factors for breast cancer, as Sun YS et al. concluded in 2017, aging, family history, reproductive factors (early menarche, late menopause, late age at first pregnancy and low parity), estrogen (endogenous and exogenous estrogens), lifestyle (excessive alcohol consumption, too much dietary fat intake, smoking) are all risk factors for breast cancer (42), the early detection and prevention of breast cancer can be promoted by increasing the overall understanding and recognition of breast cancer risk.

Relevant literature shows that the research of AI in breast cancer risk prediction is also very extensive. Nindrea RD et al. conducted a systematic review of the published ML algorithms for breast cancer risk prediction between January 2000 and May 2018, summarized and compared five ML algorithms including SVM, ANN, decision tree (DT), naive Bayes, and K-nearest neighbor (KNN) algorithms, and confirmed that the SVM algorithm was able to calculate breast cancer risk with better accuracy than other ML algorithms (43). Some studies have shown that the mammography results, risk factors, and clinical findings were analyzed and learned through an ANN combined with cytopathological diagnosis to evaluate the risk of breast cancer for doctors to estimate the probability of malignancy and improve the positive predictive value (PPV) of the decision to perform biopsy (44). Yala A and his team also developed a hybrid DL model that operates on both the full-field mammogram and traditional risk factors, and found that it was more accurate than a current clinical standard, i.e. the Tyrer-Cusick model (45). As a result, AI predicts breast cancer risk with higher accuracy than other methods, which in turn helps physicians guide high-risk populations to conduct appropriate interventions to reduce the incidence of breast cancer.

### Image Quality Improvement

Good image quality is the basis of accurate diagnoses of diseases. Image quality has a significant impact on the diagnosis rate and accuracy rate of AI for assessing breast diseases on mammography, and clear images are conducive to the detection and diagnosis of microscopic lesions. Computer algorithms for improving image quality have been proposed one after another. Because it provides more details on the data phase, directionality and shift invariance, multi-scale shearlet transform can yield multi-resolution results, which is helpful to detect cancer cells, particularly those with small contours. Shenbagavalli P and his colleagues enhanced mammogram image quality by using a shearlet transform image enhancement method and classified the DDSM database as benign and malignant with an accuracy of up to 93.45% (11). Teare P et al. used a novel form of a false color enhancement method to optimize the characteristics of mammography through contrast-limited adaptive histogram equalization (CLAHE) and utilized dual deep CNNs at different scales for classification of mammogram images and derivative patches combined with a random forest gating network, they achieved a sensitivity of 0.91 and a specificity of 0.80 (46). Image quality is the premise of an accurate diagnosis, therefore, strict image quality evaluation and improvement measures must be carried out to effectively assist radiologists and ANN systems for further analysis and diagnosis (**Table 1**).

**Table 1:** Summary of key studies on the role of AI in mammography.

n	Task	Algorithms	No. of Cases	Results	Ref.
1	detect, segment, and classify the breast masses	a completely integrated CAD system (the You-Only-Look-Once to detect, the full resolution CNN to segment, the deep CNN to recognize and classify)	112	ACC= 95.64%	(16)
2	detect, analysis, and classify microcalcifications	a deep CNN with the same 5 convolutional layers	990	ACC=89.32%	(28)
				Sen = 86.89%	
3	classify microcalcifications	an improved fisher linear discriminant analysis approach combined with a support vector machine variant	288	ACC=96%	(29)
4	segment breast masses	a hybrid method based on the active contours and fuzzy logic	57	ACC=88.08%	(32)
				Sen=91.12%	
5	detect and segment breast masses	globally supported radial basis function based collocation method	300	AUC=98%	(33)
				Sen=97.12% Spe=92.43%	
6	categorize breast density	a two-class CNN-based deep learning model	7000	AUC=94.21%	(38)
7	estimate breast cancer risk	a back-propagation learning algorithm	655	AUC=95.5% Sen=82% Spe=90%	(44)
8	enhance image quality	shearlet transform and neural network	300	ACC=93.45%	(11)

AI, artificial intelligence; CAD, computer aided diagnosis; CNN, convolutional neural network; ACC, accuracy; Sen, sensitivity; AUC, the area under the receiver operating characteristic curve; Spe, specificity.

## Applications of AI in Breast Ultrasound

As a diagnostic method with a high utilization rate, ultrasound has many advantages, such as simple operation, no radiation, and real-time operation. Therefore, ultrasound imaging has gradually become a common imaging method for the detection and diagnosis of breast cancer. To avoid a missed diagnosis or misdiagnosis caused by lack of physician experience or subjective influence and to achieve the quantification and standardization of ultrasound diagnosis, an AI system was developed to detect and diagnose breast lesions in ultrasound images (47). Related studies (48, 49) have shown that the AI systems are mainly used for the identification and segmentation of ROIs, feature extraction and classification of benign and malignant lesions in breast ultrasound imaging.

### Identification and Segmentation of ROIs

To accurately represent and diagnose the breast lesions, the lesions should first be segmented from the background. In the current clinical work, the manual segmentation of breast images was mainly carried out by ultrasound doctors, this process not only depends on the doctors’ working experience but also takes time and effort. In addition, breast ultrasound images have low contrast, blurry boundaries, and a large amount of shadows, therefore, an automatic segmentation method for breast ultrasound image lesions using AI is proposed. The segmentation process of breast ultrasound images mainly includes the detection of an ROI containing the lesion and delineation of its contours. Hu Y et al. proposed an automatic tumor segmentation method that combined a dilated fully convolutional network (DFCN) with a phase-based active contour (PBAC) model. After training, 170 breast ultrasound images were identified and segmented, and they achieved a mean DSC of 88.97%, which showed that the proposed segmentation method could partly replace the manual segmentation results in medical analysis (50). Kumar V. et al. proposed a multi-U-net algorithm and segmented masses from 258 women’s breast ultrasound images, they achieved a mean Dice coefficient of 0.82, a true positive fraction (TPF) of 0.84, and a false positive fraction (FPF) of 0.01, which are obviously better than the results with the original U-net algorithm (51). Feng Y. et al. combined a Hausdorff-based fuzzy c-means (FCM) algorithm with an adaptive region selection scheme to segment ultrasound images of breast tumors. Based on the mutual information between regions, the neighborhood around each pixel is adaptively selected for Hausdorff distance measurement. The results showed that the adaptive Hausdorff-based FCM algorithm had a better performance than the Hausdorff-based and traditional FCM algorithms (52). The identification and segmentation of lesions in breast ultrasound images saves a considerable amount of time for ultrasound physicians to quickly identify and diagnose diseases and provide a foundation and guarantee for the development of AI for automatic diagnosis of breast diseases.

### Feature Extraction

**Figure 1:**
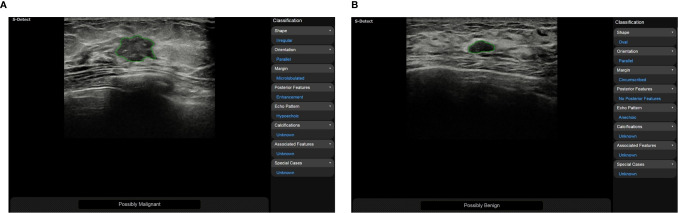
**(A)** A 50-year-old woman was diagnosed with invasive cancer, and the results of CAD (S-Detect, Samsung RS80A ultrasound system) were “possibly malignant”; **(B)** A 48-year-old woman was diagnosed with adenosis, and the results of CAD were “possibly benign”.

Ultrasound doctors usually identify and segment suspicious masses based on the morphological and texture features of the breast images. These features may be shape, orientation, edge, echo type, rear features, calcification location and hardness. Then, they classify suspicious masses according to the BI-RADS scale to quantify the degree of cancer suspicion in breast masses. The morphological features are very essential for the diagnosis of benign and malignant masses, and obtaining them correctly requires high demands on the ultrasound examiner. To reduce the dependence on the physician’s experience, AI systems have been applied to the feature extraction of breast ultrasound images. According to the research by Hsu SM et al. morphological-feature parameters (e.g., standard deviation of the shortest distance), texture features (e.g., variance), and the Nakagami parameter are combined to extract the physical features of breast ultrasound images, they classified the data using FCM clustering and achieved an accuracy of 89.4%, a specificity of 86.3%, and a sensitivity of 92.5%. Compared with logistic regression and SVM classifiers, the maximum discrimination performance of the optimal feature collection was independent of the type of classifier, indicating that the combination of different feature parameters should be functionally complementary to improve the performance of breast cancer classification (53). Zhang et al. constructed a two-layer DL architecture to extract the shear-wave elastography (SWE) features by combining feature learning and feature selection. Compared with the statistical features of quantified image intensity and texture, the results showed that the DL features had better classification performance with an accuracy of 93.4%, a sensitivity of 88.6%, a specificity of 97.1%, and an area under the receiver operating characteristic curve of 0.947 (54). Relevant studies have shown that using CAD systems (S-Detect, Samsung RS80A ultrasound system) to analyze the ultrasound features of breast masses can significantly improve the diagnostic performance of experienced and inexperienced radiologists (**Figure 1**). CAD systems may be helpful in refining breast lesion descriptions and in making management decisions, and it improves the consistency of the characteristics of breast masses among observers (49, 55).

### Benign and Malignant Classification

Breast cancer has a high incidence and mortality among women all over the world, therefore, many countries have carried out breast cancer screening for women of appropriate age. In breast disease screening, the most important thing is to distinguish breast cancer from benign breast diseases. Physicians mainly classify the lesions in breast ultrasound images based on BI-RADS. To allow doctors with different experience to reach a consistent conclusion, AI systems with benign and malignant classification functions have been developed gradually. Cirtisis A et al. classified an internal data set and an external test data set by using a deep convolution neural network (dCNN) and classified breast ultrasound images into BI-RADS 2-3 and BI-RADS 4-5. The results showed that the dCNN reached a classification accuracy of 93.1% (external 95.3%), whereas the classification accuracy of radiologists was 91.6 ± 5.4% (external 94.1 ± 1.2%). This shows that dCNNs may be used to mimic human decision making (56). Becker AS et al. used DL software to analyze 637 breast ultrasound images (84 malignant and 553 benign lesions). A randomly chosen subset of the images (n=445, 70%) was used for the training of the software, and the remaining cases (n=192) were used to validate the resulting model in the training process. The results were compared with three readers with variable expertise (a radiologist, resident, and trained medical student), and the findings showed that the neural network, which was trained on only a few hundred cases, exhibited comparable accuracy to the reading of a radiologist. There was a tendency for the neural network to perform better than a medical student who was trained with the same training data set (57). This finding indicates that the classification and diagnosis of breast diseases assisted by AI can significantly shorten the diagnostic time of physicians and improve the diagnostic accuracy of inexperienced doctors (**Table 2**).

**Table 2:** Summary of key studies on the role of AI in breast ultrasound.

n	Task	Algorithms	No. of Cases	Results	Ref.
1	segment breast tumors	a dilated fully convolutional network combined with an active contour model	170	AUC=79.5%	(50)
				ACC=71.9%	
				Sen=71.2%	
				Spe=72.6%	
2	segment breast masses	the underlying multi u-net algorithm based on CNN	433	Sen=84%	(51)
3	characterize breast tumors	fuzzy c-means clustering algorithm	160	AUC=96%	(53)
				ACC=89.4%	
				Sen=92.5%	
				Spe=86.3%	
4	detect, highlight, and classify breast lesions	deep CNN	101	AUC=83.8%	(56)
5	classify breast tumors	an industrial grade image analysis software (ViDi Suite v. 2.0)	192	AUC=98%	(57)
				Sen=97.12% Spe=92.43%	
6	classify breast tumors	a two-layer DL architecture comprised of the point-wise gated boltzmann machine and the restricted boltzmann machine	227	ACC=93.4%	(54)
				Sen=88.6%	
				Spe=97.1%	
				AUC=94.7%	
7	identify ALN involvement	DL radiomics	584	AUC=90.2%	(58)

AI, artificial intelligence; AUC, the area under the receiver operating characteristic curve; ACC, accuracy; Sen, sensitivity; Spe, specificity; CNN, convolutional neural networks; DL, deep learning; ALN, axillary lymph node.

## Applications of AI in Breast MRI

MRI is the most sensitive modality for breast cancer detection and is currently indicated as a supplement to mammography for patients at high risk (59). MRI can comprehensively evaluate the shape, size, scope and blood perfusion of breast masses through a variety of scanning sequences. However, it has disadvantages of low specificity, high cost, long examination time and selectivity for patients, therefore it is not as popularly used as mammography and ultrasound examinations. Most studies on breast imaging and DL have focused on mammography, less evidence is available concerning breast MRI (60). The study of DL in breast MRI mainly focuses on the detection, segmentation, characterization and classification of breast lesions (61–64). Ignacio Alvarez Illan et al. detected and segmented non-mass-enhanced lesions on dynamic contrast-enhanced magnetic resonance imaging (DCE-MRI) of the breast with a CAD system, and the optimized CAD system reduced and controlled the false positive rate and finally achieved satisfactory results (65). Herent P. et al. developed a DL model to detect, characterize and classify lesions on breast MRI (mammary glands, benign lesions, invasive ductal carcinoma and other malignant lesions) and achieved fine performance (60). Antropova N. et al. incorporated the dynamic and volumetric components of DCE-MRIs into breast lesion classification with DL methods using maximum intensity projection images. The results showed that incorporating both volumetric and dynamic DCE-MRI components can significantly improve CNN-based lesion classification (66). Jiang Y. et al. set up 19 breast imaging radiologists (eight academics and eleven private practices) to classify benign and malignant from DCE-MRI, and compared the classification results that only using conventionally available CAD evaluation software including kinetic maps and supplement using AI analytics through CAD software. It was found that the use of AI systems improved radiologists’ performance in differentiating benign and malignant breast lesions on MRI (67). Breast MRI is still necessary to screen patients at high risk of breast cancer. The CAD system can improve the sensitivity of examination, decrease the false positive rate, and reduce unnecessary biopsy and psychological burden of patients (68) (**Table 3**).

**Table 3:** Summary of key studies on the role of AI in breast MRI.

n	Task	Algorithms	No. of Cases	Results	Ref.
1	detect, characterize and categorize lesions	a supervised-attention model with deep learning	335	AUC=81.6%	(60)
2	classify lesions	radiomic analysis and CNN	1294	AUC=98%	(62)
3	characterize and classify lesions	the combination of unsupervised dimensionality reduction and embedded space clustering followed by a supervised classifier	792	AUC=81%	(63)
4	classify breast tumors	QuantX	111	AUC=76%	(67)
5	assess and diagnose contralateral BI-RADS 4 lesions	MRI radiomics-based machine learning	178	AUC=77%	(69)
				ACC=74.1%	
6	assess tumor extent and multifocality	CADstream software (version 5.2.8.591)	86	AUC = 88.8%	(70)
				Spe=92.1%	
				PPV=90.0%	
7	early predict pathological complete response to neoadjuvant chemotherapy and survival outcomes	linear support vector machine, linear discriminant analysis, logistic regression, random forests, stochastic gradient descent, decision tree, adaptive boosting and extreme gradient boosting	38	AUC=86%	(71)

AI, artificial intelligence; MRI, magnetic resonance imaging; AUC, the area under the receiver operating characteristic curve; CNN, convolutional neural network; BI-RADS, Breast Imaging Reporting and Data System; ACC, accuracy; CAD, computer-aided detection; Spe, specificity; PPV, positive predictive value.

## Conclusion

AI, particularly DL, is increasingly widely used in medical imaging and shows excellent performance in medical image analysis tasks. With its advantages of fast computing speed, good repeatability and no fatigue, AI can provide objective and effective information to doctors and reduce the workload of doctors and the rates of missed diagnosis and misdiagnosis (72). At present, the CAD system for breast cancer screening has been widely studied. In mammography, ultrasound, MRI and other imaging examinations, these systems can identify and segment breast lesions, extract features, classify them, estimate BD and the risk of breast cancer, and evaluate treatment effect and prognosis (39, 73–78). These systems show great advantages and potential in relieving pressure on doctors, optimizing resource allocation and improving accuracy.

## Challenges and Prospects

AI is still in the stage of “weak AI”. Although it has made rapid developments in the medical field in the past decade, it is far from the goal of being fully integrated into the work of clinicians and large-scale application in the world. At present, there are many limitations in CAD systems for breast cancer screening, such as the lack of large-scale public datasets, the dependence on ROI annotation, high image quality requirements, regional differences, and overfitting and binary classification problems. In addition, AI mostly aims for one task training and cannot solve multiple tasks at the same time, which are the challenges and difficulties that DL faces in the development of breast imaging. Meanwhile, these also provide a new impetus for the development of breast imaging diagnostic disciplines and show the broad prospect of intelligent medical imaging in the future.

In addition to their application in traditional imaging methods, CAD systems based on DL are developing rapidly in the fields of digital breast tomosynthesis (79–81), ultrasound elastography (82), contrast-enhanced mammography, ultrasound and MRI et al. (83, 84). We believe that AI in breast imaging can not only be used for the detection, classification and prediction of breast diseases, but also further classify specific breast diseases (e.g. breast fibroplasia) and predict lymph node metastasis (85) and disease recurrence (86). It is believed that with the progress of AI technology, radiologists will achieve higher accuracy with higher efficiency and more accurate classification and determination of adjuvant treatment for breast diseases to achieve early detection, early diagnosis and early treatment of breast cancer and benefit the majority of patients.

## Author Contributions

MY, M-HY, JY, and W-ZL contributed to the collection of relevant literature. S-EZ, JL, and CFD contributed significantly to literature analysis and manuscript preparation. Y-ML sorted out the literature and wrote the manuscript. JY provided a lot of help in the revision of the manuscript. H-RY and X-WC were responsible for design of the review and provided data acquisition, analysis, and interpretation. All authors contributed to the article and approved the submitted version.

## Funding

This work was supported by the key research and development project in Hubei Province(2020BCB022), the Joint Fund project of Hubei Provincial Health and Family Planning Commission (WJ2018H0104), the Natural Science Foundation of Hubei Province (2019CFB2876), and Science and Technology Bureau of Shihezi, China (No. 2019ZH11).

## Conflict of Interest

The authors declare that the research was conducted in the absence of any commercial or financial relationships that could be construed as a potential conflict of interest.
